# The New York City Typhoid Epidemic of 1913

**Published:** 1914-06

**Authors:** 


### Abstracts and Selections.

### The New York City Typhoid Epidemic of 1913.

The Central Council of Public Health of the city of New York made an investigation of the typhoids of 1913, with a view of aiding the Department of Health in its crusade against this disease. Their report (*N. Y. Med. Jour.*, Jan. 10, 1914) confirmed the conclusions already published to the effect that most of the cases were contracted from infected foods mostly milk, and a small number from close contact with the sick as in the case of a mother nursing a son. Incidentally they say that only one-third of the cases in the country as a whole are water borne. Their conclusions and recommendations are so important to all the country that we quote them in full:

SUMMARY.

1. Typhoid fever is acquired by drinking and eating food contaminated by the excreta of typhoid patients. Water, milk, vegetables, oysters and shellfish, are the chief articles of food by means of which typhoid fever germs are carried into the stomach.
2. The comparison of the typhoid death rates of 2.5 per 100,000 population in Hamburg, 3.6 in Berlin, 4.0 in London, 4.1 in Vienna, and 6.7 in Paris with the typhoid death rates of 11.6 in New York, 11.6 in Boston, 13.7 in Chicago, 17.4 in Philadelphia, and 23.2 in Washington, D. C., strikingly indicates how much more there remains to be done along the lines of prevention of this disease in this country.

3. A large percentage of typhoid outbreaks is milk borne and preventable. The recent serious outbreak in Manhattan (autumn, 1913) had its origin in contaminated milk. This fact again emphasizes the need for efficient pasteurization of all raw and certified milk.

4. The Department of Health of the City of New York is not equipped financially to control efficiently the production and distribution of the milk sold in this city.

5. The Department of Health has used its present force of typhoid inspectors to good advantage.

6. A large proportion of cases of typhoid in this city seems to have originated through careless contact with typhoid patients. This is probably the result of:

A. The incomplete reporting of typhoid cases by physicians and hospitals and the consequent insufficient exercise of control by the Department of Health.

B. This apparent lack of appreciation on the part of physicians and the public of the contagiousness of the disease; and

C. The inadequacy of the educational methods of the Department of Health concerning sanitary precautions, food preparation and disposal of excreta, and the lack of follow up visits by health department inspectors to ascertain whether instructions given have been carried out.

RECOMMENDATIONS.

The Central Council of Public Health of the City of New York recommends:

1. That the Department of Health take more rigorous action in enforcing the law concerning the reporting of typhoid fever cases.

2. That the attention of the boards of managers of hospitals be called to the fact that hospitals frequently fail to report cases of typhoid fever and that an appeal be made to them for prompt and complete reporting of all such cases.

3. That the Department of Health require the hospitals to enter cases of typhoid fever in the special books provided by the Department of Health for the registration of cases of tuberculosis and venereal diseases.

4. That the Department of Health be urged to enforce rigorously its rules and regulations with respect to sterilization of milk cans and milk bottles.

5. That the health department's force of inspectors, both for typhoid and milk inspection, be increased and that the medical and sanitary corps of the department be made more flexible; and that in the case of milk supervision special attention be paid to the inspection of the milk at the receiving stations.

6. That the Department of Health be requested to institute supplementary visits in typhoid cases and to require from its inspectors reports on the continued observance of sanitary rules in the homes of the patients.

7. That the Department of Health exert its influence in the matter of early sending to hospitals those cases of typhoid fever in which the department's inspectors report inadequate sanitary care.

8. That the Department of Health consider the revision of its circulars of instruction with the view of making them still more helpful to the public.

9. That the county medical societies of this city be asked to stimulate interest among their members as to the importance and necessity of prompt reporting of typhoid fever cases and of adequate isolation and disinfection.

10. That greater use be made by physicians of the facilities of the Henry Street Settlement in securing adequate disinfection and proper isolation in cases of typhoid; and

11. That the attention of the public be called to the resolution adopted by the New York Academy of Medicine urging that all persons in an infected family and any person who has been exposed in any way to the disease follow up the sanitary precautions usually taken in such cases, subjecting themselves to immunization at the hands either of their private physician or of the Department of Health.

